# A retrospective analysis of negative diffusion-weighted image results in patients with acute cerebral infarction

**DOI:** 10.1038/srep08910

**Published:** 2015-03-17

**Authors:** Lian Zuo, Yue Zhang, Xiahong Xu, Ying Li, Huan Bao, Junjie Hao, Xin Wang, Gang Li

**Affiliations:** 1 Department of Neurology, East Hospital, Tongji University, Shanghai 200120, China; 2 Department of Neurology, Zhongshan Hospital, Fudan University, Shanghai 200032, China

## Abstract

We aimed to investigate the clinicoradiologic determinants of negative diffusion-weighted image (DWI) results in patients with acute cerebral infarction (ACI). The medical records were reviewed of ACI patients. Patients were divided to the DWI positive and negative group. Positive DWI was used as independent variable and patients' clinicoradiologic factors were used as co-variables for multivariate logistic regression analysis. 349 patients received initial cerebral MRI within 72 hours of admission. Lacunar infarction was most common (42.1%) followed by posterior circulation infarction (30.1%) and partial anterior circulation infarction (18.1%). The majority of the patients (72.2%) had an NIHSS score of less than 5 at admission. 316 patients (90.54%) were positive on initial DWI. Patients with smoking, initial SBP ≥ 140 or DBP ≥ 90 mmHg, initial fasting plasma glucose (FPG) ≥7.0 mmol/L, initial MRI from onset of disease >1 d and anterior circulation infarction were liable to show positive DWI. Furthermore, DWI negative patients had significantly lower NIHSS scores (IQR 0,1,2) than DWI positive patients (IQR 1,2,4) (*P* = 0.000) at two weeks post onset of acute cerebral infarction. In conclusion, multiple clinicoradiologic factors are associated with negative and positive DWI and further delineation of these factors is required in future prospective studies.

Acute cerebral infarction is a highly debilitating or lethal disease and its early diagnosis closely relies on magnetic resonance imaging (MRI) study^1^. Diffusion-weighted image (DWI) can detect the presence of infarct in the early stage of brain infarction and is reported to have a sensitivity of 88% to 100% for acute cerebral infarction^2^. The American Academy of Neurology Guideline considers DWI helpful in finding acute cerebral infarct (grade A) and reliable for early diagnosis of acute cerebral infarction and believes that the method can be used to guide thrombolysis^3^. Currently, DWI is widely accepted as the de-facto clinical reference standard for infarct core lesion. However, many scholars have found that some acute cerebral infarction patients exhibit negative DWI^4^,^5^,^6^,^7^, which hampers clinical diagnosis. This phenomenon is worthy of further investigation. Rosso *et al.* reviewed the imaging data of patients within 6 hours of of observed symptom onset of anterior circulation infarction by 3.0 T and 1.5 T MR and found the sensitivity and specificity and accuracy of 1.5 T DWI were superior to 3.0 T DWI, suggesting that the intensity of magnetic field of MR instruments impacts on DWI of cerebral infarction during the acute phase^8^.

Therefore, to help to clarify whether the clinicoradiologic factors of patients with acute cerebral infarction had any effect on DWI results during the acute phase, we retrospectively analyzed data of patients with acute cerebral infarction examined using 3.0 T MRI to exclude the disturbance of the intensity of magnetic field.

## Methods

### Patients

We retrospectively reviewed the clinical and radiological data of patients with acute cerebral infarction who were hospitalized at the Department of Neurology, East Hospital, Tongji University, Shanghai, China, between January 1 and December 31, 2013. A patient was eligible for inclusion in the study 1) if he or she was aged > 18 years, 2) had received a diagnosis of acute cerebral infarction and received initial cerebral MRI within 72 hours of admission. Cerebral infarction was diagnosed by neurologists according to the 2013 AHA/ASA guideline by 1) the presence of ischemic lesion compatible with the pathologic and imaging characteristics of the vasculature in the central nervous system (CNS) or the presence of clinical evidence for ischemic injury of the CNS, 2) the presence of neurological deficits lasting more than 24 hours due to ischemic lesions confirmed on conventional MRI of the brain, and 3) the exclusion of other CNS diseases^9^,^10^. A cerebral CT scan or MRI was performed to exclude patients who had other intracranial pathologies (e.g., tumor, infection). Patients who received recent vascular stenting, cardiac valve replacement and pace maker placement or patients with other conditions such as unstable vital signs rendering them unfit for MRI were excluded. The study was approved by the local institutional review board at East Hospital, Tongji University and patient consent was not required due to the retrospective nature of this study. Patient information was anonymized and re-identified prior to analysis.

### Patient evaluation

All patients were studied at admission as baseline evaluation. Patients' age, gender, time from symptom onset to first MRI, severity of neurological deficit on admission and stroke cause were retrieved. History of hypertension, diabetes, atrial fibrillation, smoking, and stroke was obtained. Initial blood pressure (BP) was measured on admission. Routine biochemical studies were done for fasting plasma glucose (FPG), glycated hemoglobin, initial hemoglobin, low density lipoprotein (LDL), and homocysteine. Severity of neurological deficit was evaluated at admission and two weeks post onset using the National Institutes of Health Stroke Scale (NIHSS), a 15-item scale that measures the level of neurologic impairment (scores range from 0 to 42, with higher scores indicating increasing severity). Examiners were trained stroke neurologists. Clinical classification was performed by trained stroke neurologists within one week after stroke onset using the Oxfordshire Community Stroke Project (OCSP) classification^11^ and TOAST classification.

### Imaging studies

MRI was done using Philips 3.0 T scanner. Cerebral MRI was done using T1WI, T2WI, DWI, and FLAIR. Scan was done with a slice thickness of 6 mm and at a slice interval of 1 mm with DWI b value = 0, 1000 s/mm^2^. The scan parameters are shown in Table 1. Patients with negative DWI on initial MRI were re-examined one week after initial cerebral MRI, and the imaging data from the two examinations were compared to determine the presence of culprit lesions in combination with clinical manifestations. Patients were divided to the DWI positive and negative group according to initial DWI findings. The radiological data were interpreted by two equally experienced radiologists with more than 15 years of experiences in neuroimaging and there was no difference between their interpretations.

### Therapeutic intervention

All patients with acute cerebral infarction received thrombolytic therapy within 3–4.5h and patients with posterior infarction received such therapy within 6 h of onset after exclusion of contraindications. Patients who were not indicated for thrombolysis were given antithrombotic and anticoagulation therapy.

### Statistical analysis

Data were expressed as 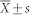 or interquartile range (IQR) and analyzed using SPSS 17.0. Univariate analysis was done first and data were analyzed using Student's *t* test or Mann-Whitney U test. Chi square test or Fisher exact test was used for numerical data. Variables with *P* < 0.15 in the univariate analysis was entered into multivariate analysis. In addition, OCSP and TOAST classification, and baseline NIHSS scores were entered into multivariate logistic regression analysis using positive DWI as independent variable and the clinicoradiologic factors were used as co-variables. *P* < 0.05 was considered statistically significant.

## Results

### Patient demographic and baseline characteristics

**Figure 1:**
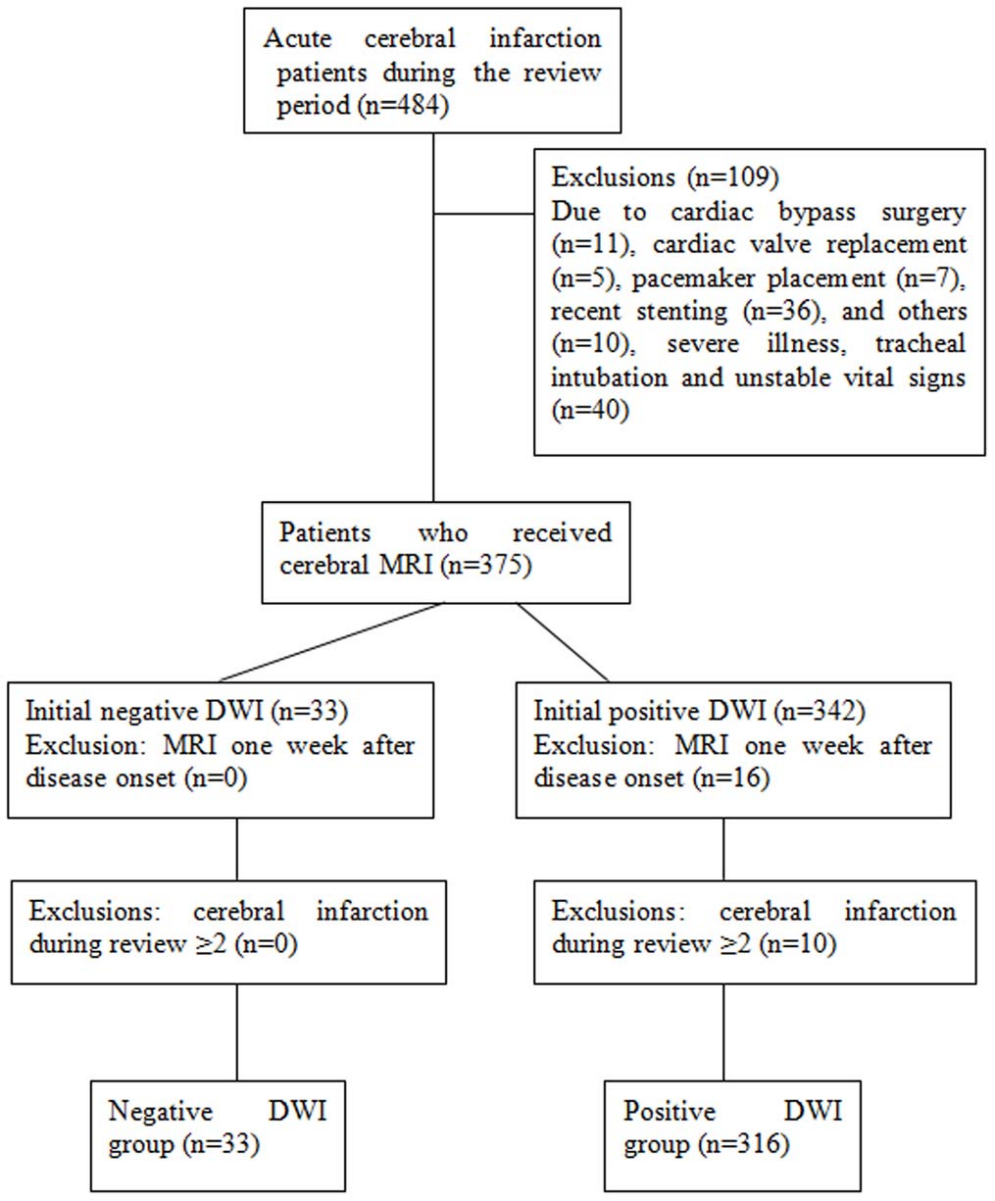
The study flowchart.

Four hundred and eighty-four patients were hospitalized because of acute cerebral infarction during the review period and 349 of them received initial cerebral MRI within 72 hours of admission. The study flowchart is shown in Fig. 1 and patient demographic and baseline characteristics are listed in Table 2. The time to initial cerebral MRI from onset of disease was 24 hours or less in 18.3% of the patients. Lacunar infarction was most common in these patients (42.1%) followed by posterior circulation infarction (30.1%) and partial anterior circulation infarction (18.1%). The majority of the patients (72.2%) had an NIHSS score less than 5 at admission.

### Characteristics of DWI positive and negative patients with acute cerebral infarction

Three hundred and sixteen (90.54%) patients were positive and 33 (9.46%) were negative on initial DWI. Their demographic and baseline data are shown in Table 2. A greater proportion of DWI positive patients were male (63.6% vs. DWI negative, 45.5%; *P* > 0.05). A greater proportion of patients had higher initial BP (77.8% vs. DWI negative, 66.7%; *P* > 0.05) and initial FPG (26.9% vs. DWI negative 9.1%; *P* < 0.05) in patients with positive DWI. A higher proportion of patients who were DWI negative underwent initial cerebral MRI within 24 hours of disease onset (30.3% vs. DWI positive, 17.1%; *P* > 0.05). Though a smaller percentage of DWI positive patients had an NIHSS score less than 5 at admission (71.2%), there was no statistical difference compared to DWI negative patients (81.8%; *P* > 0.05). DWI negative and positive patients were comparable in other demographic and baseline characteristics (Table 2). According to the OCSP classification, approximately half (51.5%) of DWI negative patients had lacunar infarction followed by posterior circulation infarction (42.4%) (Table 3). Similarly, DWI positive patients, 41.1% had lacunar infarction followed by (posterior circulation infarction (28.8%). According to the TOAST classification, 60.6% of DWI negative patients (vs. 41.8% in DWI positive patients) had small artery occlusion followed by large artery arteriosclerosis (33.3% vs. 46.7% in DWI positive patients).

**Figure 2:**
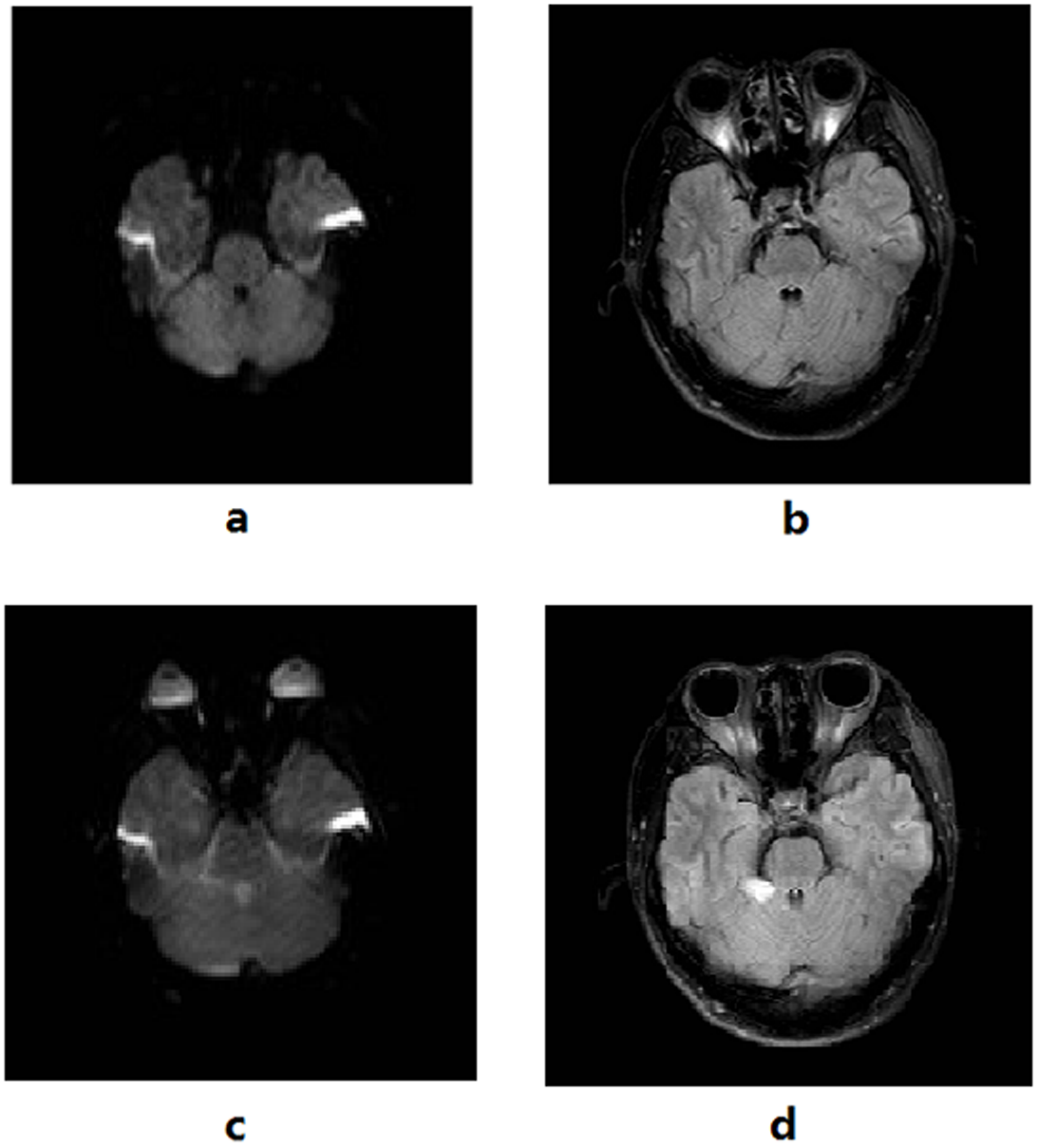
A 25-year old man with acute cerebral infarction received MRI within 2.5 hours of onset. His NIHSS score was 3 and MRI failed to reveal any positive findings. The right superior cerebellar artery was not visualized on cerebral MRA. The patient was stared with thrombolytic therapy and MRI after one week showed patchy ischemic infarct in the right cerebellum. Initial cerebral MRI (a) and FLAIR (b) at 2.5 hours after onset; repeat DWI (c) and FLAIR (d) at one week.

**Figure 3:**
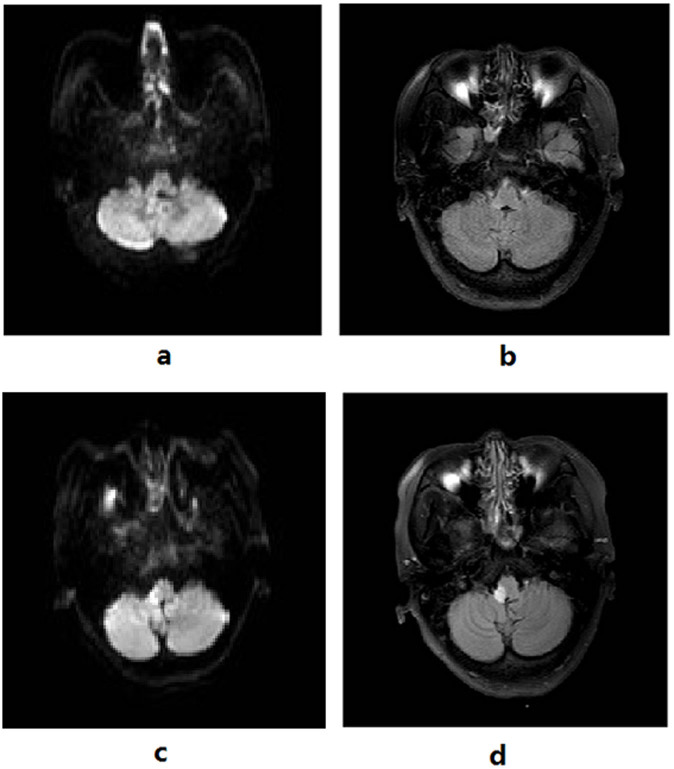
A 44-year old woman with acute cerebral infarction had a NIHSS score of 5 at admission. No acute lesion was seen on MRI DWI at admission and cerebral MRA indicated indistinct visualization of the right vertebral artery. Cervical MRA showed narrowing of the right vertebral artery, stenosis of V2–V5. Cerebral MRI after one week indicated infarction of the right medulla oblongata. Initial cerebral MRI DWI (a) and FLAIR (b); repeat DWI (c) and FLAIR (d).

Moreover, five (15.2%) patients who were DWI negative initially showed positive findings upon reexamination one week later; three patients showed lesions on both DWI and FLAIR while two patients showed lesions on FLAIR only (Fig. 2 and 3).

### Risk factors for positive DWI findings upon initial cerebral MRI

Our univariate analysis showed that smoking (*P* = 0.026), baseline BP (*P* = 0.148), baseline FPG (*P* = 0.025), LDL (*P* = 0.037) and time to initial cerebral MRI (*P* = 0.062) were significantly different between DWI positive and negative patients (Table 2). These factors were further used as covariates for multivariate logistic regression analysis using positive DWI as an independent variable. We found that history of smoking was associated with a 2.8-fold increase in the risk for positive DWI (*P* = 0.0196; OR: 2.772; 95%CI, 1.177–6.525) (Table 4). In addition, initial SBP ≥ 140 or DBP ≥ 90 mmHg was associated with an approximate 2-fold increase in the risk for positive DWI (*P* = 0.0398, OR: 2.240; 95%CI, 1.038–4.831). Furthermore, initial FPG ≥ 7.0 mmol/L was associated with more than 3 fold increase (*P* = 0.0416, OR: 3.805; 95%CI, 1.052–13.762) in the risk for positive DWI. Moreover, initial MRI from onset of disease > 1 d was associated with an approximately 3 fold increase in the risk for positive DWI (*P* = 0.0053, OR: 3.080; 95%CI, 1.396–6.795) were risk factors for positive DWI (Table 4). Anterior circulation infarction (*P* = 0.0122, OR: 13.858; 95%CI, 1.776–108.154) was associated with the greatest (more than 14-fold) increase in the risk for positive DWI findings compared with posterior circulation infarction.

### NIHSS scores of DWI negative and positive patients

DWI negative and positive patients had comparable baseline NIHSS scores at admission [DWI negative, IQR(1,2,5) vs. DWI positive, IQR(1,3,5); *P* = 0.13] (Table 5). At two weeks post onset of acute cerebral infarction, DWI positive patients had significantly higher NIHSS scores than DWI negative patients [DWI negative, IQR(1,1,2) vs. DWI positive, IQR(1,2,4); *P* = 0.000].

## Discussion

### Physiopathologic mechanisms of DWI

Cerebral DWI is a sensitive method for acute cerebral infarction. Positive DWI lesions appear within several minutes to several hours of the onset of acute cerebral infarction^12^,^13^,^14^. The brain maintains apparently intact morphology when perfusion is reduced to 30% of normal though neurons may partially lose certain function. If perfusion is returned to normal, no abnormality will be detected by cerebral MRI. However, if hypoperfusion worsens and cerebral blood flow is further reduced to 15–20% of normal, the function of membrane Na-K-ATPase is impaired, leading to disequilibrium of ions across the membrane and the development of cytotoxic edema^15^. High intensity signal appears in DWI early thanT1W, T2W and FLAIR. Negative DWI on initial cerebral MRI in acute cerebral infarction patients has been reported, but most of them are anecdotal case reports^4^,^5^,^6^,^7^. In our study, we used 3.0 T, which avoids the impact of magnetic field strength on DWI. Rosso *et al.*^8^ reviewed the imaging data of patients within 6 hours of anterior circulation infarction by 3.0 T and 1.5 T MR and found that 3.0 T DWI had a higher false positive rate. However, unlike our study, they only included patients with anterior circulation infarct and no patients with posterior infarction or lacunar infarction. Here, our retrospective analysis of 349 patients showed that close to one in ten acute cerebral infarction patients (9.5%) showed negative DWI on initial cerebral MRI. This is higher than the rate (5.8%) reported by Oppenheim *et al.*^4^, but lower than that (25.6%) reported by Sylaja *et al.*^16^. Time to initial cerebral MR was >3 hours from onset for our patients; patients who were within the time window for thromoblysis therapy were DWI positive or DWI negative. Those who were DWI negative on initial cerebral MR still received thromoblysis after contraindications were ruled out. Because these patients' symptoms persisted less than 24 hours, it remains uncertain whether they had transient ischemic attack (TIA). It has been reported that positive DWI in TIA patients correlated with symptoms that persist ≥60 min and the more prolonged the symptoms were, the greater the likelihood of permanent pathological injury was 17,18. This is also the reason for modified definition of TIA. For those who had initial cerebral MR within 24 hours of disease onset and were still DWI negative on repeat cerebral MR, we believe that these patients had cerebral infarction as their neurologic deficits persisted more than one week. For these patients, perfusion weighted imaging may help define cerebral infarction or TIA. In addition, Qiao *et al.* reported that spin labeling may help identify perfusion defects in TIA patients^19^.

### Clinical correlates of initial positive DWI in patients with actue cerebral infarction and the underlying mechanisms

We found that, compared to DWI negative patients, a greater proportion of DWI positive patients were male, had higher initial BP and initial FPG. In addition, a markedly greater proportion of DWI positive patients had a lower rate of posterior circulation infarction, but a higher rate of anterior circulation infarction. DWI negative and positive patients were comparable in other demographic and baseline characteristics. Our multivariate logistic regression analysis showed that history of smoking, initial SBP ≥ 140 or DBP ≥ 90 mmHg, initial FPG ≥ 7.0 mmol/L, initial MRI from onset of disease >24 hours and anterior circulation infarction were associated with markedly increased risk for positive DWI.

We revealed it is interesting that smoking markedly increased the risk of positive DWI on initial cerebral MRI (*P* < 0.05). Smoking is known to be closely associated with ischemic stroke. Abbruscato *et al*. reported that the activity of Na-K-2Cl cotransporter is increased to maintain ion equilibrium across the cells in the blood brain barrier when the activity of Na-K-ATPase is decreased as occurs during cerebral ischemia^20^. Paulsonet *et al.* showed that nicotine markedly decreased the activity of Na-K-2Cl cotransporter, leading to cellular toxic edema^21^, which may explain why cerebral infarction patients with a history of smoking are at increased risk for positive DWI.

It has been further shown that increased FPG at admission is a negative predictor for the outcome of acute ischemic stroke patients^22^,^23^. Our multivariate logistic analysis showed no statistical difference in our DWI negative and positive patients in history of diabetes, and glycated hemoglobin, but revealed that initial FPG ≥ 7.0 mmol/L was a risk factor for positive DWI in acute cerebral infarction patients. Though a similar proportion of acute cerebral infarction patients had a history of hypertension in the DWI negative and positive group, we found that higher initial BP (≥140/90 mmHg) was a risk factor for positive cerebral DWI. Clinical and animal studies showed that higher initial FPG induces damages against the vascular endothelium and increases permeability of blood brain barrier, consequently enlarging the size of the infarct and aggravating edema^24^,^25^,^26^. Hou *et al* found that high BP increased the amount of G-strophanthin analogues in the brain that inhibit Na^+^K^+^ATPase^27^. We speculate that these could be one of the causes that higher initial FPG and BP are risk factors for positive DWI in acute cerebral infarction patients.

### Effect of time to initial cerebral MRI and infarct location on DWI

We found that site of infarction and time to initial cerebral MRI correlated with negative DWI in acute cerebral infarction patients. Our multivariate analysis showed that time to initial cerebral MRI is an important risk factor for positive DWI with time to initial MRI within 24 hours of disease onset more likely to yield negative DWI lesions. This is consistent with the findings by Oppenheim et al. who found that negative DWI finding correlated with time to initial MRI examination in patients with acute cerebral infarction^4^. The earlier the time to initial cerebral MRI, the more likely it is to obtain negative DWI findings. Five (15.2%) of our patients who were initially DWI negative showed positive findings upon reexamination one week later.

Current literature indicates negative DWI on initial cerebral MRI also correlates with site of cerebral infarction; Negative DWI lesions are seldom reported in patients with anterior circulation infarction^6^. In the current study, 2 patients who were initially DWI negative had anterior circulation infarction and they received initial cerebral MRI within 4.5 hours of disease onset. Our multivariate logistic regression analysis revealed that anterior circulation infarction patients are more likely to show positive DWI than posterior circulation infarction patients. Posterior circulation infarction patients may exhibit imaging abnormalities in DWI later in the acute phase compared with other sites of infarction. Periodic reexamination may help yield positive DWI findings. Posterior circulation infarction exhibits imaging abnormalities in DWI later than infarction at other sites^28^,^29^,^30^,^31^. Infarct is small in the posterior circulation and may not become detectable even on repeat cerebral MRI. It has been reported that there was a gap in time between the development of neuronal dysfunction due to focal ischemia and water diffusion difficulty, which may cause DWI delay. Another contributor to delayed DWI positivity is inadequate signal to noise ratio in the early stage of the disease or MR artifacts interfering with DWI. Some investigators speculated about the presence of a second ischemic episode^4^,^5^,^6^,^7^,^28^,^29^,^30^,^31^. This hypothesis, however, is not supported by the findings of the current study which showed improvement of neurological function of DWI negative patients between two MRI examinations.

Sylaja *et al.* reexamined DWI negative patients 30 days after initial MRI of acute cerebral infarction patients and found that a portion of these patients were still DWI negative even though hyperintense signal was observed in FLAIR^16^. Some of our DWI negative patients classified with lacunar infarction and posterior circulation infarction who did not receive thrombolytic therapy showed negative MRI findings even though their symptoms persisted more than 24 hours. Therefore, for DWI negative patients, prompt and periodic reexamination by MRI may identify the culprit lesion in acute cerebral infarction patients.

### Future directions and limitations

Mismatch between DWI and perfusion weighted imaging (PWI) is widely believed to be an indication of salvageable tissue after an infarction^35^. The DWI abnormality is typically smaller than the PWI abnormality, and the region where both are abnormal is regarded as infarct core, and unsalvageable. The area where PWI is abnormal but DWI is normal, the penumbra, is typically salvageable. In DWI negative patients, a PWI lesion might be visible to highlight the area of infarction. Furthermore, this could indicate that this area is not permanently damaged and that reperfusion would strongly benefit this area. This is a commonly used measure in stroke imaging^33^. In this retrospective study, PWI was not performed when DWI was carried out and therefore we could not determine whether there was a mismatch between PWI and DWI. This is a limitation of the current study. Cho *et al.* reported that 3 patients with acute cerebral anterior circulation infarction showed negative DWI on cerebral MR while PWI indicated the presence of perfusion defect^34^. Fustier *et al.* reviewed DWI negative patients on initial cerebral MRI who received thromoblysis therapy and found that these patients had better prognosis than DWI positive patients^35^. Diffusion-perfusion mismatch helps determine early whether patients can benefit from thromoblysis therapy^36^. In DWI negative patients, the following scenarios may occur if PWI is undertaken: 1) there is a total mismatch between the two (PWI+ and DWI−). Ischemia is demonstrable on one hand while there are no positive DWI findings on the other hand after prompt reperfusion. 2) PWI and DWI are both negative, which is frequently seen in migraine and lasts for 1–48 hours^32^. Straka *et al*. reported a real time evaluation system which can detect the diffusion-perfusion mismatch in 5 to 7 minutes^33^, which may facilitate our exploration of the causes and mechanisms of negative DWI.

## Conclusion

In conclusion, we confirmed that negative DWI is associated with time to initial cerebral MRI after disease onset and site of infarction. The earlier the time to initial cerebral MRI is, the more likely a negative DWI is. Furthermore, posterior circulation infarction patients are more likely to have negative DWI than anterior circulation infarction patients. Patients with history of smoking, higher initial BP and FPG are more likely to have positive DWI lesions. However, the current study is limited by the retrospective nature of the analysis and the conclusion may not be applicable to primary healthcare settings as it was carried out at a single tertiary care institution. In addition, even though patients were included who received initial cerebral MRI within 72 hours of admission, great variation may exist in time to initial cerebral MRI due to variation in time to seek medical care after disease onset. Furthermore, the majority of our patients had NIHSS < 5 and had mild stroke and the number of DWI negative patients was relatively low. Another limitation of the current study is the lack of a gold standard for the ultimate diagnosis of acute cerebral infarction even though DWI negative patients in the study met the clinical definition for cerebral infarction. This will be addressed in our future studies by using PWI or adjusting b value in DWI. Therefore, the findings of the current study need to be confirmed by prospective studies with more clearly defined time to initial cerebral MRI from disease onset.

## Author Contributions

L.Z., Y.Z., X.W. and G.L. designed of the experiments; L.Z., G.L., X.X., Y.L., H.B. and J.H. collected the data; G.L., X.W., L.Z., Y.Z., J.H. and X.X. conducted the experiment; X.W., G.L. and L.Z. wrote the main manuscript text and all authors reviewed the manuscript.

## Figures and Tables

**Table 1:** MRI scan parameters used in the current study

	T1W	T2W	FLAIR	DWI	MRA
Matrix	176 × 141	256 × 163	240 × 134	112 × 89	396 × 228
Resolution	0.449 mm	0.449 mm	0.449 mm	1.8 mm	0.357 mm
FOV	230 × 183	230 × 183	230 × 183	230 × 230	200 × 200
TR	15 ms	1494 ms	11000 ms	1360 ms	25 ms
TE	4.6 ms	80 ms	120 ms	57 ms	3.5 ms
TI			2800 ms		
Acquisition time	56.4 s	35 s	1 min39 s	16 s	3 min12 s

TR: repetition time; TE: echo time; TI: inversion time; FOV: field of view.

**Table 2:** Univariate analysis of determinants of positive DWI in acute cerebral infarction patients undergoing initial cerebral MRI

Variables	Negative	Positive	P
Gender, n			0.306
Male	15	201	
Female	18	115	
Age, years	66.97 ± 12.92	68.26 ± 11.80	0.602
~50	2	20	
50 ~ 60	6	59	
60 ~ 70	8	85	
70 ~ 80	13	93	
80~	4	59	
Hypertension, n			0.356
Yes	26	225	
No	7	91	
Diabetes, n			0.243
Yes	7	98	
No	26	218	
Smoking, n			0.026
Yes	8	140	
No	25	176	
Atrial fibrillation, n			0.75
Yes	2	24	
No	31	292	
Prior stroke, n			0.496
Yes	6	74	
No	27	242	
Initial blood pressure, n			0.148
SBP ≥ 140 mmHg or DBP ≥ 90 mmHg	22	246	
SBP < 140 mmHg or DBP < 90 mmHg	11	70	
Initial FPG (mmol/L), n			
<7.0 mmol/L	30	231	0.025
≥7.0 mmol/L	3	85	
HbA1c, n			
<6.5%	23	198	0.425
≥6.5%	10	118	
LDL, n			
≤2.6 mmol/L	30	236	0.037
>2.6 mmol/L	3	80	
Homocysteine, n			
≤15.4 μmol/L	27	276	0.373
>15.4 μmol/L	6	40	
Initial Hb, n			
Male ≥ 120 g/L female ≥ 110 g/L	30	265	0.287
Male < 120 g/L female < 110 g/L	3	51	
Baseline NIHSS, n			
<5	27	225	0.195
≥5	6	91	
Time to initial cerebral MRI (d), n			
≤1	10	54	0.062
>1	23	262	

**Table 3:** OCSP and TOAST classification of DWI positive and negative patients

Variables	Negative	Positive
OCSP^a^		
Total anterior circulation infarction	1	33
Partial anterior circulation infarction	1	62
Lacunar infarction	17	130
Posterior circulation infarction	14	91
TOAST^b^		
Large artery arteriosclerosis	11	148
Small artery occlusion	20	132
Cardiac emboli	2	30
Unknown or others	0	6

^a^*P* = 0.013, lacunar infarction vs. anterior circulation infarction; *P* = 0.006, posterior circulation infarction vs. anterior circulation infarction; *P* = 0.634, lacunar infarction vs. posterior circulation infarction.

^b^*P* > 0.05 for all between group comparison.

**Table 4:** Multivariate logistic regression analysis of determinants of positive DWI in acute cerebral infarction patients undergoing initial cerebral MRI

Variables	SE	Wald	OR	95%CI	*P*
**History of smoking**	0.4368	5.4475	2.772	1.177–6.525	0.0196
**Initial SBP ≥ 140 or DBP ≥ 90 mmHg**	0.3923	4.2247	2.240	1.038–4.831	0.0398
**Initial FPG ≥ 7.0 mmol/L**	0.6559	4.1506	3.805	1.052–13.762	0.0416
**LDL > 2.6 mmol/L**	0.4593	3.1097	0.445	0.181–1.094	0.0778
**NIHSS score at admission >5**	0.5326	0.4690	1.440	0.507–4.090	0.4934
**Initial MRI from onset of disease >1 d**	0.4037	7.7643	3.080	1.396–6.795	0.0053
**Anterior vs. posterior infarction**	1.0483	6.2881	13.858	1.776–108.154	0.0122
**Cardiac emboli vs. small artery**	0.8568	0.7482	2.098	0.391–11.249	0.3871

**Table 5:** NIHSS scores of DWI negative and positive patients at admission and two weeks post onset of acute cerebral infarction

	All patients	DWI negative	DWI positive	*P*
**All**	349	33	316	
**NIHSS score at admission**				
Mean(SD)	3.65(3.652)	2.82(3.06)	3.74(3.70)	
IQR	1,2,5	1,2,3	1,3,5	0.130
Range	0,21	0,15	0,21	
**NIHSS score at two weeks**				
Mean(SD)	2.73(3.171)	1.18(2.53)	2.9(3.22)	
IQR	1,2,4	0,1,2	1,2,4	0.000*
Range	0,17	0,11	0,17	
**Difference between NIHSS score at admission and week 2**				
Mean(SD)	0.92(2.705)	1.64(2.737)	0.84(2.695)	
IQR	0,0,2	0,1,2	0,0,2	0.013*
Range	−14,15	0,15	−14,14	

*P<0.05.
